# Supplementary material for: Mentality and behavior in COVID-19 emergency status in Japan: Influence of personality, morality and ideology
Source: PLoS One. 2020 Jul 10;15(7):e0235883. doi: 10.1371/journal.pone.0235883 (PMC7351180; doi:10.1371/journal.pone.0235883)
Supplement: Attachment — Submitted filename: PO_Response_01_R05_QK.docx [file pone.0235883.s002.docx]

**In the following point-by-point response, the comments from reviewers are given in Italics, and the line numbers are based on the file labeled 'Revised Manuscript with Track Changes'.**

***

**Response to Reviewer 1**

**Comments:** *Thanks for addressing all my comments. However, some responses were not yet sufficient to convince the reviewer that this manuscript is ready for publication.*

*I am not yet convinced by the rational for this study. The response says “our results could provide empirical evidence to the governance, and help them to implement certain general measures to reduce people’s tendency of neuroticism and improve their conscientiousness and agreeableness”*

*This still sounds that personalities can be changed by the government.*

*Moreover, it is claimed that “by encouraging people to take their own responsibility in preventing infection and epidemic in their daily life and to better understand and express gratitude to the efforts of other people”.*

*I do not think that government can change people’s personalities by those exercises. In addition, the government is still encouraging people to exercise these precautions, REGARDLESS their personalities.*

*Thus, it is not clear to me how the results of these studies can help the government. If the aim of this study is just to explore the relationships between variables, that would significantly reduce the value of the study. As I suggested, it would be better to at least include the follow up data should the results of this study cannot help the government and society.*

**Response:** Thank you for your comments and concerns. To be very clear, we do not consider that the government should change people’s personalities. Considering your comments, we revised the relevant descriptions by emphasizing the minds and behaviors associated with neuroticism, conscientiousness, and agreeableness (Line 407-414). We also revised the passage regarding governance and explained that the government should focus on personality traits as well as personality-related thinking and behaviors, rather than focusing on specific individuals or groups of individuals who display prominent personality traits of concern (Line 414-423). Furthermore, please consider that the implications to governance were not only about personality (Line 407-423) but also concerned morality, ideology, and comparisons based on social demographic data (Line 424-445). The implications from the viewpoint of personality was just one aspect of this paragraph.

We hope we have made the above points clear with the minor revision. With respect to the follow-up data, as mentioned in our last reply, we are now collecting longitudinal data through weekly wave surveys. The COVID-19 pandemic remains active. Thus, we would like to publish the longitudinal data as a different paper. The purpose of this study was to provide immediate data collected at the beginning of the outbreak.
